# Comparison of patients with small (≤2 cm) breast cancer according to adherence to breast screening program

**DOI:** 10.1371/journal.pone.0186988

**Published:** 2017-11-02

**Authors:** Jung Min Park, Soong Jun Bae, Changik Yoon, Hye Sun Lee, Hak Woo Lee, Sung Gwe Ahn, Seung Ah Lee, Joon Jeong

**Affiliations:** 1 Department of Surgery, Gangnam Severance Hospital, Yonsei University College of Medicine, Seoul, Republic of Korea; 2 Biostatistics Collaboration Unit, Yonsei University College of Medicine, Seoul, Republic of Korea; 3 Department of Surgery, CHA Bundang Medical Center, CHA University, Seongnam, Republic of Korea; University of North Carolina at Chapel Hill School of Medicine, UNITED STATES

## Abstract

**Background:**

We investigated whether adherence to breast screening would yield a clinical benefit even among patients with small breast cancer (≤2 cm) by comparing differences between those who did and did not adhere to breast screening.

**Methods:**

Patients who were diagnosed with invasive T1 breast cancer and treated at Gangnam Severance Hospital from January 2006 to June 2014 were included. Of the 632 study patients, 450 and 182 were classified as screen-adherent and non-adherent. Adherence to the breast screening program was defined as the completion of breast screening examinations within 3 years before cancer diagnosis. Recurrence-free survival (RFS) and metastasis-free survival (MFS) were compared between the groups. Propensity score matching were applied to compare survival outcome.

**Results:**

Adherent patients were more likely to have a lower histologic grade (*P* < 0.001), high estrogen receptor expression (*P* = 0.040), and lower HER2-positivity (*P* = 0.026). The adherent group had more favorable subtypes compared to the non-adherent group, with a greater percentage of Luminal/HER2-negative subtype (66.7% vs. 56.5%) and a lower percentage of HER2 subtype (8.3% vs. 16.7%). The RFS and MFS were significantly better in the adherent group (*P* = 0.003, 0.010, respectively). In the case-matched cohort, superior survival of the adherent group was maintained.

**Conclusions:**

Adherence to breast screening in patients with small breast tumors was associated with more favorable tumor biology and better prognosis. Our findings suggest that adherence to breast screening might offer clinical benefits in terms of tumor biology as well as early detection.

## Introduction

The goal of cancer screening is early tumor detection, followed by appropriate treatment. Regular screening mammography for breast cancer has been widely recommended and is supported by solid evidence: most population-based randomized controlled trials have reported reduced mortality among women who undergo screening mammography [1–5]. As in other developed countries, a national health screening program in the Republic of Korea initiated a population-based mammography screening program in 1999 [6].

Breast tumors detected by mammography screening are known to be smaller than those detected outside of screening [7–11]; accordingly, better prognosis is largely observed in populations wherein cancer is detected through screening programs. Of note, studies have also shown that mammographic cancer detection is associated with a better prognosis than that of similarly sized tumors found outside of screening [7–9,11–17]. These studies suggest that breast screening itself could be considered a favorable prognostic variable independent of the primary tumor size, axillary metastasis, age at cancer diagnosis, and tumor grade. Additionally, even among patients with ductal carcinoma in situ (DCIS), those with screening-detected tumors had better outcomes than did those with symptom-detected tumors [18].

In this study, we investigated whether adherence to a breast screening program would yield clinical benefit even among patients with small invasive breast cancers, as outcomes might be less affected by the detection method. We compared the pathological and biological differences, including the proportions of immunohistochemistry (IHC)-based subtypes, between groups of patients who did and did not adhere to a screening program.

## Materials and methods

### Study population

The institutional review board of Gangnam Severance Hospital, Yonsei University, Seoul, Korea, approved this study, which was conducted in accordance with good clinical practice guidelines and the Declaration of Helsinki. The need for informed consent was waived because of the retrospective design, under the approval of the institutional review board. Patients with T1 breast cancer who were treated at Gangnam Severance Hospital between January 2006 and June 2014 were identified. We excluded patients with bilateral breast cancer and those who were treated with neoadjuvant chemotherapy. Women younger than age 40 years were also excluded because general population screening was recommended from age 40 years. Raw data of these patients is provided online (S1 Data).

### Tumor detection method

Physicians in the Breast Cancer Center of Gangnam Severance Hospital interviewed patients and documented information about the tumor detection method in the medical charts. Adherence to the breast screening program was defined as the completion of breast screening examinations within 3 years prior to the diagnosis of breast cancer because the Korean national health screening program offers biennial mammography screening. The completion of private mammography was also considered adherence to the breast screening program. Patients who had never participated in a breast screening program or completed screening ≥3 years before a diagnosis of breast cancer were classified as non-adherent. The patients for whom information about the breast screening program was not available were considered unclassified.

With a consideration of mammographic density which affects results of screening program, we included mammographic density category evaluated by Breast Imaging Reporting and Data System (BI-RADS).

### Immunohistochemistry markers

For our IHC study, we stained formalin-fixed, paraffin-embedded tissue sections obtained from surgical specimens using appropriate antibodies specific for four markers: estrogen receptor (ER; 1:100 dilution, clone 6F11; Novocastra, Newcastle upon Tyne, UK), progesterone receptor (PR; clone 16; Novocastra, UK), human epidermal growth factor receptor 2 (HER2; 4B5 rabbit monoclonal antibody; Ventana Medical Systems, Tucson, AZ, USA), and Ki-67 (MIB-1; Dako, Glostrup, Denmark). Patients were stratified by ER and PR IHC test results into four groups using the modified Allred system: strong, Allred score 7–8; moderate, Allred score 5–6; weak, Allred score 2–4; and negative, Allred score 0–1 [19]. The HER2 status was defined as positive with a score of 3+ and negative with a score of 0 or 1+ [20]. Tumors with scores of 2+ were sent for fluorescent in situ hybridization (FISH) analysis, according to the protocol given by the supplier (PathVysion kit; Vysis, Downers Grove, IL, USA or HER2 inform; Ventana). Ki67 expression was measured by an experienced pathologist and reported as a percentage of positive tumor cells (range: 0–100%).

### IHC-based subtype

Tumors were classified into four molecular subtypes based on ER, PR, HER2 expression: luminal-HER2 negative (ER+ and/or PR+, HER2-), luminal-HER2-positive (ER+ and/or PR+, HER2+), HER2 (ER-, PR-, HER2+), and triple-negative breast cancer (TNBC; ER-, PR-, HER2-).

### Statistical analysis

Continuous variables such as age and tumor size were compared by Mann–Whitney U test. Discrete variables were compared using the chi-square test. Recurrence-free survival (RFS) was measured from the date of the first curative surgery to the date of the first loco-regional recurrence or distant metastasis. Metastasis-free survival (MFS) was measured from the date of the first curative surgery to the date of the first distant metastasis. The Kaplan–Meier method was used to estimate the RFS and MFS, and the estimated survival curves were compared using the log-rank test. Using Harrell *c*-statistic, the concordance index (*c*-index) was calculated to measure the concordance for time-to event data, in which increasing values between 0.5 and 1.0 indicated improved prediction. The Cox’s regression-hazard model was used for univariate and multivariable survival analyses.

To adjust for potential confounding factors, we performed an individual propensity score-matching method using a Greedy algorithm in which randomly selected individuals in the adherent group were paired with comparable individuals in the non-adherent group. The one control per one case was selected based on tumor size, lymph node metastasis, histologic grade, ER, and HER2.

All analyses were performed using SPSS version 18 (SPSS; Chicago, IL, USA) and SAS (version 9.4, SAS Inc., Cary, NC, USA). Statistical significance was defined as a P-value < 0.05 or a 95% confidence interval (CI) that did not include 1.

## Results

### Comparisons of clinical and pathologic characteristics

A total of 684 patients were included; of these, 450, 182, and 52 were categorized as screen-adherent, non-adherent, and unclassified, respectively. We further compared the clinical and pathological characteristics between the adherent and non-adherent groups (Table 1). Patients who adhered to the breast screening were more likely to have smaller tumors (*P* < 0.001) than were the non-adherent patients. Although 490 patients (77.5%) had node-negative disease, the non-adherent group was more likely to have a higher nodal stage (*P* = 0.004). The non-adherent patients had more advanced stage disease (*P* < 0.001), higher histologic grade (*P* < 0.001), higher ER-negative rate (*P* = 0.040), and higher HER2 positive rate (*P* = 0.026). Regarding adjuvant treatments, the adherent group was more likely to receive breast-conserving surgery *(P* < 0.001) and endocrine therapy (*P* = 0.007), and less likely to receive chemotherapy (*P* < 0.001).

**Table 1:** Clinical and pathologic characteristics according to the screen-adherence.

	Original Cohort			Case-matched cohort		
Variables	Screening-adherence (n = 450)	Screening-non-adherence (n = 182)	*P*-value	Screening-adherence (n = 137)	Screening-non-adherence (n = 137)	*P*-value
**Age, median (range)**	51 (40–87)	49 (40–82)	0.055	50 (40–84)	49 (40–82)	0.462
**Tumor size, median (range)**	1.2 (0.1–2.0)	1.5 (0.3–2.0)	<0.001	1.5 (0.3–2.0)	1.5 (0.3–2.0)	0.872
**Mammographic density** ^a^^,^ ^b^			0.094			0.348
I	11 (2.7)	2 (1.2)		4 (3.1)	1 (0.8)	
II	51 (12.4)	32 (18.6)		16 (12.5)	22 (17.1)	
III	286 (69.8)	120 (69.8)		91 (71.1)	93 (72.1)	
IV	62 (15.1)	18 (10.5)		17 (13.3)	13 (10.1)	
**T stage**			<0.001			0.878
T1a	47 (10.4)	6 (3.3)		6 (4.4)	6 (4.4)	
T1b	115 (25.6)	24 (13.2)		19 (13.9)	22 (16.1)	
T1c	288 (64.0)	152 (83.5)		112 (81.8)	109 (79.6)	
**N stage**			0.004			0.802
0	356 (79.1)	133 (73.1)		103 (75.2)	106 (77.4)	
N1	89 (19.8)	38 (20.9)		32 (23.4)	30 (21.9)	
N2	4 (0.9)	8 (4.4)		2 (1.5)	1 (0.7)	
N3	1 (0.2)	3 (1.6)				
**Stage**			<0.001			0.988
I	366 (81.3)	136 (74.7)		109(79.6)	108 (78.8)	
II	80 (17.8)	35 (19.2)		26 (19.0)	27 (19.7)	
III	4 (0.9)	11 (6.0)		2 (1.5)	2 (1.5)	
**Grade** ^a^			<0.001			0.881
I or II	373 (86.9)	126 (72.0)		110 (80.3)	108 (78.8)	
III	56 (13.1)	49 (28.0)		27 (19.7)	29 (21.2)	
**Estrogen receptor** ^c^			0.040			0.398
Positive	334 (74.2)	120 (65.9)		95 (69.3)	92 (67.2)	
Negative	116 (25.8)	62 (34.1)		42 (30.7)	45 (32.8)	
**Progesterone receptor** ^c^			0.471			0.623
Positive	280 (62.2)	107 (58.8)		84 (61.3)	79 (57.7)	
Negative	170 (37.8)	75 (41.2)		53 (42.3)	58 (42.3)	
**HER2**			0.026			0.352
Negative	351 (81.2)	122 (72.6)		115 (83.9)	108 (78.8)	
Positive	81 (18.8)	46 (27.4)		22 (16.1)	29 (21.2)	
**Ki67** ^a^			0.194			1.000
≥20	87 (19.4)	44 (24.2)		32 (23.4)	32 (23.4)	
<20	362 (80.6)	138 (75.8)		105 (76.6)	105 (76.6)	
**Surgery**			<0.001			0.182
BCS	287 (63.8)	87 (47.8)		81 (59.1)	69 (50.4)	
TM	163 (36.2)	95 (52.2)		56 (40.9)	68 (49.6)	
**Chemotherapy**			<0.001			0.146
Not given	248 (55.1)	70 (38.7)		70 (51.1)	57 (41.6)	
Given	202 (44.9)	111 (61.3)		67 (48.9)	80 (58.4)	
**Endocrine therapy**			0.007			0.256
Not given	83 (18.4)	52 (28.6)		28 (20.4)	37 (27.0)	
Given	367 (81.6)	130 (71.4)		109 (79.6)	100 (73.0)	
**Radiotherapy**			0.040			0.394
Not given	170 (37.8)	85 (46.7)		56 (40.9)	64 (46.7)	
Given	280 (62.2)	97 (53.3)		81 (59.1)	73 (53.3)	

^a^ Missing value

^b^ Mammographic density was categorized according to Breast Imaging Reporting and Data System

^c^ Positive, Allred score 2–8; Negative, Allred score 0–1.

Abbreviations: HER2, human epidermal growth factor receptor 2; BCS, breast-conserving surgery; TM, total mastectomy

Moreover, we described our two groups in relation to the presence of symptoms (S1 Fig). All of the non-adherent had positive symptoms, while 161 (35.8%) of the adherent had positive symptoms. Details of symptoms in relation to the screening-adherence were presented in S1 Table.

### Comparisons for IHC-based subtypes

**Fig 1:**
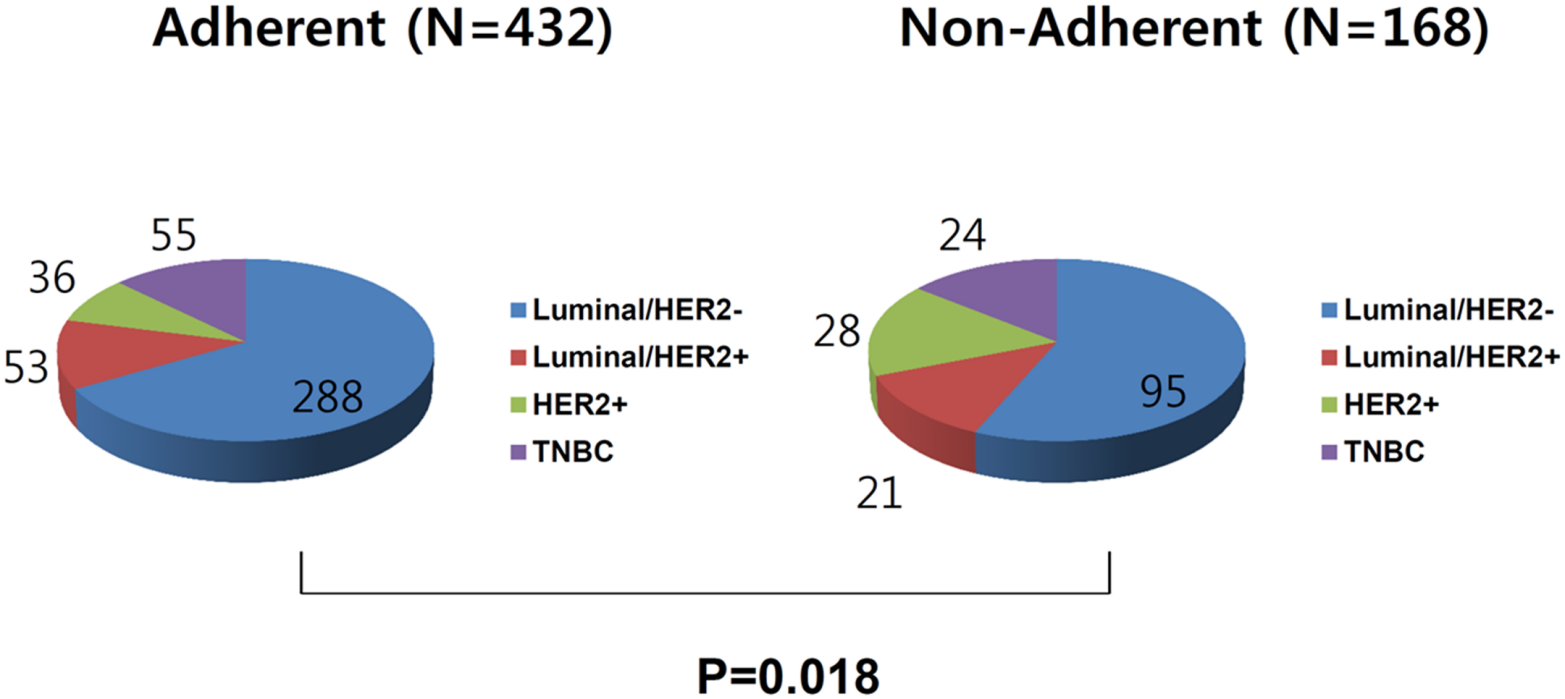
Comparisons of immunohistochemistry (IHC)-based subtypes between groups that did or did not adhere to breast screening. Screening-adherent patients more frequently presented with a favorable subtype (66.7% vs. 56.5% for Luminal/HER2-negative), and less frequently presented with an aggressive subtype, compared with non-adherent patients (8.3% vs. 16.7% for HER2).

In intergroup comparisons of IHC-based subtype frequencies, the adherent group had more favorable subtypes compared to the non-adherent group, with a greater percentage of Luminal/HER2-negative subtype (66.7% vs. 56.5%) and a lower percentage of HER2 subtype (8.3% vs. 16.7%; Fig 1).

### Clinical outcomes

**Fig 2:**
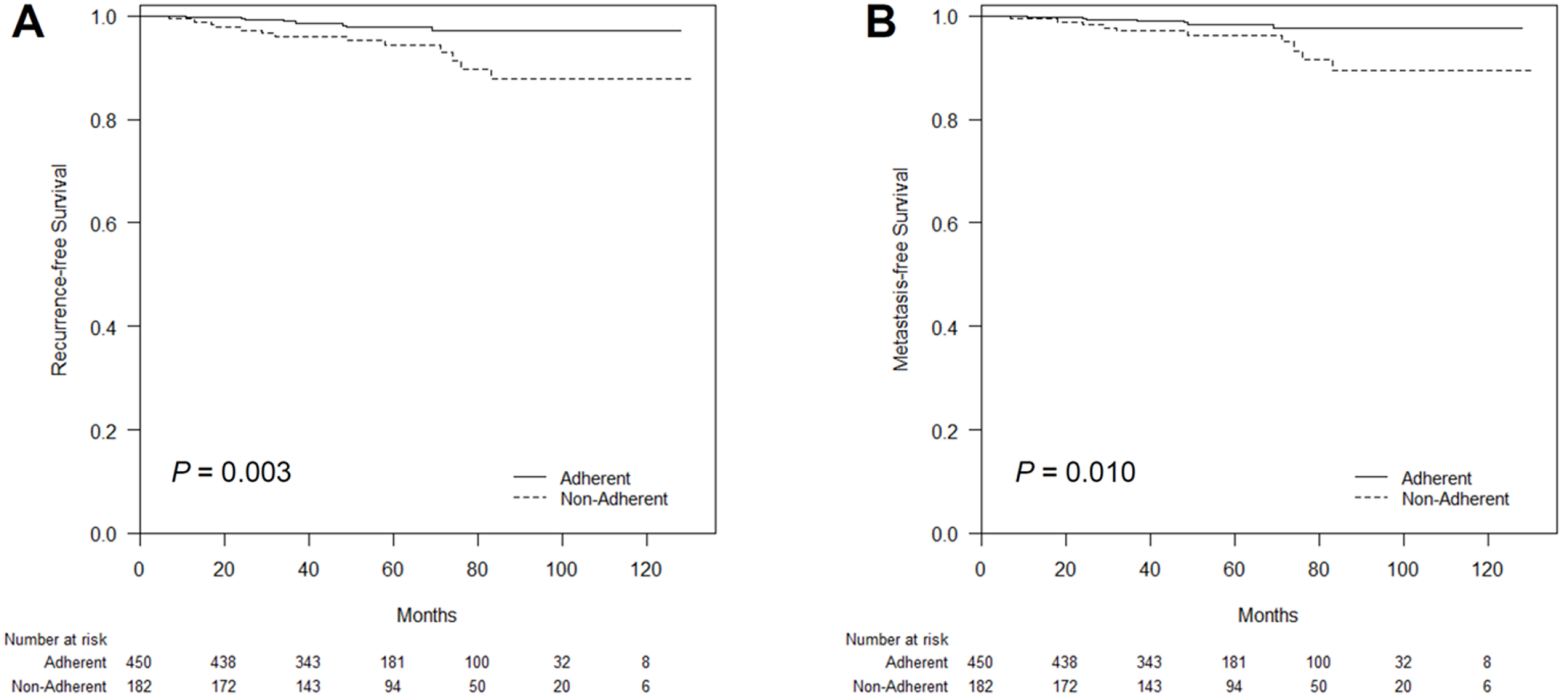
Kaplan–Meier plots of recurrence-free survival (RFS) and metastasis-free survival (MFS) according to adherence to breast screening. (A) The RFS and (B) MFS differed significantly according to the adherence status (*P* = 0.003 and *P* = 0.010, respectively, log-rank test).

During a median follow-up period of 59 (range: 12–131) months, there were 22 recurrences occurred, including 5 loco-regional and 17 distant metastases. The RFS and MFS differed significantly according to adherence to the breast screening program based on the log-rank test (*P* = 0.003 and *P* = 0.010, respectively; Fig 2).

To demonstrate the adherence to screening as an independent prognostic factor, we conducted our analyses in two ways. First, multivariate models for RFS and MFS were constructed using the Harrell *c*-statistic. In a multivariate analysis using a Cox regression hazard model, adherence to breast screening (adjusted hazard ratio: 2.974; 95% CI: 1.280–6.909) was identified as a prognostic factor for RFS, independent of the nodal status, tumor size, nodal status, grade, ER status (Table 2). In a multivariate model for MFS, the adherence showed a marked trend toward a significant prognostic factor (adjusted hazard ratio: 2.464; 95% CI: 0.876–6.932).

**Table 2:** Multivariate survival analyses of recurrence-free survival and metastasis-free survival.

Variables	Recurrence-free survival		Metastasis-free survival	
	Multivariate (*P*)	Hazard ratio (95% CI)	Multivariate (*P*)	Hazard ratio (95% CI)
**T stage**	0.762		0.796	
T1a /b		Reference		Reference
T1c		1.195 (0.379–3.766)		0.853 (0.255–2.849)
**N stage**	0.717		0.260	
Negative		Reference		Reference
Positive		1.196 (0.452–3.164)		1.814 (0.644–5.106)
**ER**	0.442		0.277	
Positive		Reference		Reference
Negative		1.569 (0.497–4.949)		2.814 (0.534–8.933)
**Histologic grade**	0.460		0.763	
I and II		Reference		Reference
III		1.1.536 (0.492–4.793)		1.239 (0.308–4.977)
**Adherence**	0.033		0.087	
Adherence		Reference		Reference
Non-Adherence		2.974 (1.280–6.909)		2.464 (0.876–6.932)

Multivariate *P* values: Cox regression hazard model

Abbreviations: ER, estrogen receptor; HER2, human epidermal growth factor receptor 2

**Fig 3:**
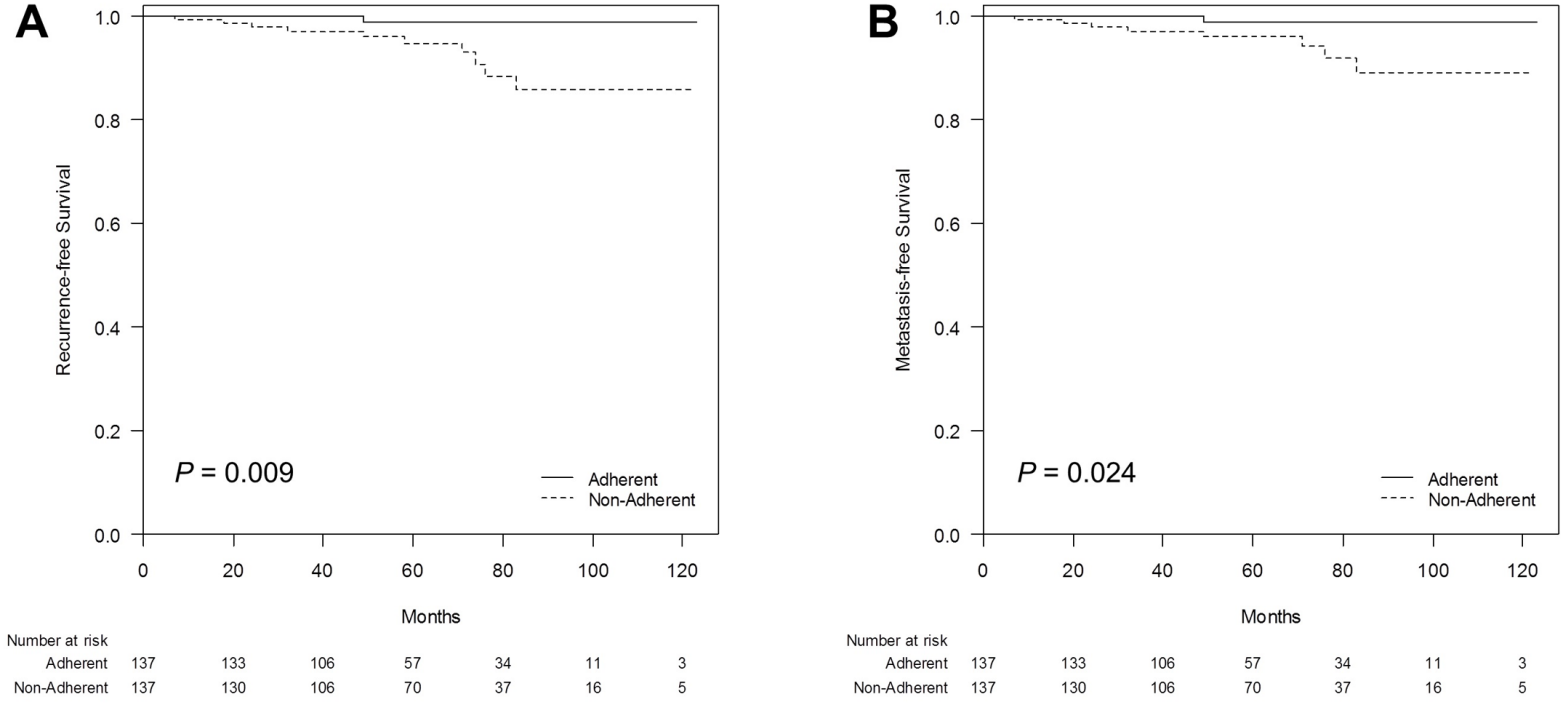
Kaplan–Meier plots of recurrence-free survival (RFS) and metastasis-free survival (MFS) according to adherence to breast screening in the case-matched cohort. (A) The RFS and (B) MFS differed significantly according to the adherence status (*P* = 0.009 and *P* = 0.024, respectively, log-rank test).

In addition, to adjust for potential confounding factors including adjuvant treatments, we adopted case-matching method, and assigned 137 patients for each group. Of the 274 patients in the propensity-matched cohorts, tumor characteristics and adjuvant treatments were not different according to the adherence to screening (Table 1). In the matched cohort, 12 women showed recurrences, with 2 loco-regional and 10 distant recurrences. Improved RFS and MFS of the adherent group was still observed (*P* = 0.009 for RFS, *P* = 0.024 for MFS; Fig 3).

## Discussions

Even in this modern era characterized by advances in systemic therapy, screening mammography for the detection of early-stage breast cancers remains associated with a reduction in breast cancer-related mortality [5,21]. In our selected group of patients with small tumors, who were selected to circumvent the effects of early detection via screening programs, the positive effect of breast screening was substantiated in terms of tumor biology and clinical outcome. We found that when compared with tumors diagnosed outside of a breast screening program, those detected via screening had more favorable biologic characteristics and, consequently, a better prognosis, despite the small tumor size (≤2 cm).

Previous studies have shown a link between screening-detected tumors and a less aggressive biological profile, including a lower histologic grade and mitotic count, strong ER and PR expression, reduced HER2 expression, and a lower cell proliferation rate, compared with symptom-detected tumors. In support of these findings, another study used a novel tumor genotyping approach to demonstrate that symptom-detected tumors have higher copy number gains, compared with screen-detected tumors [22]. Likewise, our findings regarding the better biologic characteristics of tumors diagnosed via breast screening were concordant with findings from previous studies.

A previous study of DCIS compared the subtype frequencies among screen-detected tumors and symptom-detected tumors [18]. In that study, favorable subtypes were more frequent among screen-detected DCIS cases, compared with symptom-detected cases. In accordance with this study, we found that the adherent group had a higher rate of luminal/HER2-negative and a lower rate of TNBC, compared with the non-adherent group.

A lower rate of chemotherapy given could be addressed as another advantage of the adherence to breast screening (45.4% in the adherence vs.62.3% in the non-adherence). In our study, the patients with adherence to breast screening showed a better outcome than the patients with non-adherence despite of a lower rate of adjuvant chemotherapy. It is reasonable because the non-adherence group had higher rates of HER2 expression and TNBC subtype. The higher chance of sparing toxicity from chemotherapy in the adherence group would be recognized as the benefit of breast screening program.

We recognize that our study had some limitations. One major caveat is the retrospective design, as well as the inclusion of a small number of patients from a single institute. Future studies involving multi-institutional databases are warranted to affirm our results. Also, another limitation is to acknowledge that clinical outcome can be affected by various variables including duration of endocrine treatments and complexity of clinical behavior, which are not fully considered in our analyses. Thus, our findings should be carefully integrated into current knowledge on pro and cons of breast screening program. Despite these limitations, one strength of our study is the population included in the study, which represents patients treated in daily practice because the definition of breast screening included private practice breast screening, as well as the national screening program. Furthermore, our findings obtained among selected patients with small, early-phase tumors further support the use of breast screening, as tumors detected using this modality remained associated with better tumor biologic characteristics and a favorable prognosis.

## Conclusions

Breast cancers identified under adherence to breast screening are associated with a more favorable tumor biology and better prognosis, even during the early phase. Our findings suggest that adherence to breast screening, in addition to providing early detection, may yield clinical benefits due to more favorable tumor biology and outcomes.

## Supporting information

S1 Data Raw data. (XLSX)

S1 Fig Consort diagram in relation to the presence of symptoms. (TIF)

S1 Table Details of symptoms in relation to the adherence to screening program. (DOCX)
