# Supplementary figures and images for: Comparison of patients with small (≤2 cm) breast cancer according to adherence to breast screening program
Source: PLoS One. 2017 Nov 2;12(11):e0186988. doi: 10.1371/journal.pone.0186988 (PMC5667799; doi:10.1371/journal.pone.0186988)

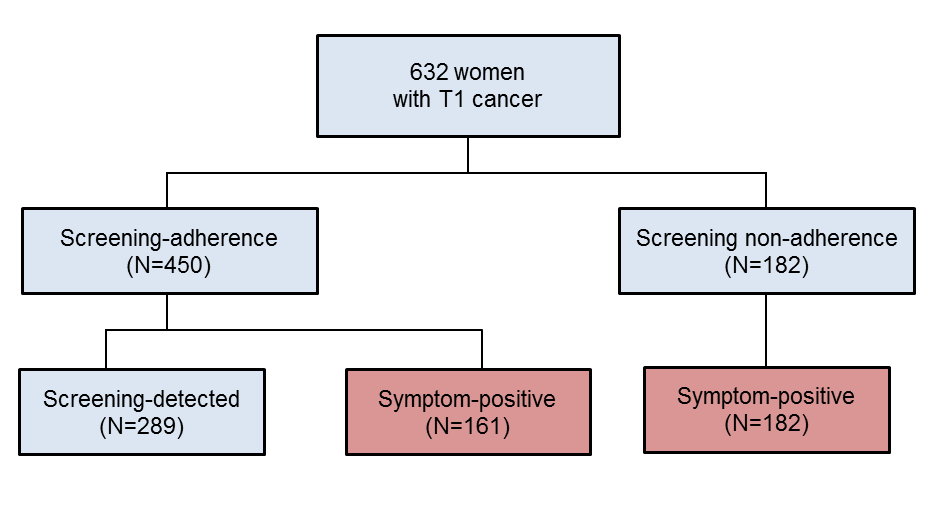

Supplement: S1 Fig — (TIF) [file pone.0186988.s002.tif]
