# Supplementary material for: Comparison of patients with small (≤2 cm) breast cancer according to adherence to breast screening program
Source: PLoS One. 2017 Nov 2;12(11):e0186988. doi: 10.1371/journal.pone.0186988 (PMC5667799; doi:10.1371/journal.pone.0186988)
Supplement: S1 Table — (DOCX) [file pone.0186988.s003.docx]

**S1 Table. Details of symptoms in relation to the adherence to screening program**

| **Kind of Symptoms** | **Adherence (n=450)** | **Non-adherence (n=182)** | **P-value** |
| --- | --- | --- | --- |
| Palpability | 135 (30.0) | 160 (87.9) | <0.001 |
| Nipple discharge | 8 (1.8) | 5 (2.7) | 0.536 |
| Skin change | 8 (1.8) | 4 (2.2) | 0.751 |
| Pain | 30 (6.7) | 28 (15.4) | 0.001 |
| Others | 1 (0.2) | 1 (0.5) | 0.493 |
